# Monitoring of Regional Ventilation Distribution Using Electrical Impedance Tomography in Pediatric Patients With Chest Physiotherapy—A Feasibility Study

**DOI:** 10.1002/ppul.71014

**Published:** 2025-02-26

**Authors:** Johanna Moersdorf, Thomas Muders, Christian Putensen, Ulrike Heller, Andreas Mueller, Lukas Schroeder

**Affiliations:** ^1^ Department of Neonatology and Pediatric Intensive Care Medicine University Children´s Hospital Bonn Bonn Germany; ^2^ Department of Anesthesiology and Intensive Care Medicine University Hospital Bonn Bonn Germany; ^3^ Center for Physiotherapy and Physical Therapy, University Hospital Bonn Bonn Germany

**Keywords:** chest physiotherapy, children, electrical impedance tomography (EIT), regional ventilation

## Abstract

**Background:**

Current evidence remains unclear whether pediatric patients with acute or chronic lung diseases benefit from chest physiotherapy (CPT) during spontaneous breathing. Electrical impedance tomography (EIT) offers the opportunity to assess treatment effects of CPT on regional ventilation distribution.

**Methods:**

We conducted a prospective feasibility study between 10/2023 and 05/2024. Pediatric patients with need for active or passive CPT were screened. EIT measurements were performed immediately before CPT intervention (T1), and within 30 min after CPT intervention (T2).

**Results:**

Overall, 25 patients were enrolled, with two patients enrolled twice at different admissions, resulting in a total of 54 EIT‐measurements. The mean horizontal center of ventilation (CoVx) and the mean vertical CoVy were equally distributed at T1 and T2, without any difference seen when subdividing for mode of CPT. The mean global inhomogeneity index (GI) decreased from 0.38 to 0.36 (∆GI: ‐6%, *p* = 0.939) in the overall cohort, with a significant decrease between T1 and T2 in patients with active (∆GI: ‐10%, *p* = 0.015), but not in patients with passive CPT (∆GI: +6%, *p* = 0.199). In patients with a localized pulmonary finding in the radiological assessment (*n* = 10) we observed an increase in the ventilated lung area (EIT‐pixel) of the affected lung side after CPT (T1: 141 vs. T2: 176; *p* = 0.128).

**Conclusion:**

EIT seems feasible to monitor changes in regional ventilation distribution after CPT in pediatric patients. Patients with localized pulmonary radiological findings and patients after active CPT appear to benefit most from CPT, whereas there is a subset of individuals with no response to CPT.

## 1 Introduction

Pediatric patients suffering from acute or chronic pulmonary diseases might benefit from chest physiotherapy (CPT), including passive or active physiotherapeutic interventions. CPT is widely used in daily clinical routine in the pediatric population, including preterm infants, term infants, toddlers and children up to high school age. Despite the wide use of passive and active CPT the evidence for CPT is still under debate and is controversial, depending on the underlying disease and age of the infants. Multiple review studies or editorials published in the last decade are reflective of this debate [1, 2, 3, 4, 5], illustrating both positive and negative data after CPT interventions. Electrical impedance tomography (EIT) has entered the stage of available radiation‐free diagnostic tools in the pediatric population in the last decade. EIT is a non‐invasive, radiation‐free procedure, which can be performed at bedside with an elastic belt placed around the thorax to monitor regional ventilation distribution and regional lung mechanics. EIT measures changes in the electrical conductivity of tissues. By applying electrical currents to the body surface and measuring the resulting voltage distribution of different tissues, regional differences in the bio‐impedances of aerated lung‐tissue due to ventilation can be detected and transferred in a color‐scaled image [6, 7]. In two adult studies (one feasibility study and one randomized controlled trial) EIT was identified as a feasible monitoring tool in patients with CPT intervention [8, 9]. Furthermore, recent studies in the pediatric field described EIT as an innovative and new monitoring technique for patients with CPT, as well as a monitoring tool during mechanical ventilation [10, 11]. Both studies include data from pediatric patients during mechanical ventilation. Nevertheless, there is still a gap in research focusing on EIT for regional ventilation monitoring in pediatric patients with the need for CPT for acute or chronic lung disease. In particular, to date there are no available data evaluating EIT in spontaneously breathing pediatric patients with CPT.

The main objective of this study was to investigate the feasibility of EIT for the measurement of regional ventilation distribution in spontaneously breathing pediatric patients, and to evaluate whether EIT can depict changes in lung aeration before and after CPT.

## 2 Material and Methods

### 2.1 Study Population, Inclusion and Exclusion Criteria

All pediatric patients with acute or chronic lung diseases admitted to the Children´s Hospital during the study period from 10/2023 to 05/2024 were eligible for study participation. Inclusion criteria were as follows: acute or chronic lung disease with need for passive or active CPT (see more detailed description below) as prescribed by the attending physician, body weight > 3.5 kg, and/or thoracic circumference ≥ 36 cm. Patients were allowed to be supported with non‐invasive respiratory support (oxygen supply via nasal cannula, continuous positive airway pressure [CPAP] via bi‐nasal prong or mask, or non‐invasive ventilation via full‐face mask). The exclusion criteria for EIT measurements were as follows: (a) all patients with invasive mechanical ventilation (as this will be part of a separate study); (b) patients with palliative care, (c) patients with unstable cardiopulmonary conditions making EIT‐measurements impossible; (d) EIT belt could not be placed accurately due to a small thoracic circumference; (e) surgical wound dressings or thoracic drainages leading to an inadequate skin contact of EIT electrodes; (f) implanted cardiac pacemaker (according to the manufacturer's guidelines of the EIT‐device).

### 2.2 Ethical Study Approval and Guidelines

Patients were prospectively enrolled in the study after informed written consent was obtained from the parents or legal representative. The study was approved by the local ethics committee with the study number 236‐23EP, and the study was registered in a WHO acknowledged clinical trial register with the trial number DRKS00032506. The methods used for clinical research were performed in accordance with the STROBE (strengthening the reporting of observational studies in epidemiology) guidelines and in accordance with the Declaration of Helsinki.

### 2.3 Patients’ Demographics and Pulmonary Diseases

All patients with an acute or chronic pulmonary disease due to (a) viral, bacterial, or fungal infections, (b) genetic syndromes/inherited diseases resulting in hypopnea and respiratory insufficiency, (c) condition after prolonged mechanical ventilation (> 24 h), or (d) patients at risk for chronic pulmonary disease and where CPT was performed as prophylaxis (condition after transplantation or immunodeficiency), were potential candidates for study inclusion. When a chest X‐ray or lung ultrasound was performed during the in‐hospital stay, these data were retrospectively screened, and medical reports of chest X‐rays as well as lung ultrasounds were evaluated.

### 2.4 EIT‐Measurements

EIT‐data were collected and analyzed using a PulmoVista500 device (Dräger Medical GmbH, Lübeck, Germany), using a frame rate of 50 Hz. The 16‐electrode belt was placed around the thorax at the axilla level and in patients < 10 kg directly under the axilla (belt‐size 4XS and 3XS). To ensure the fitting and adequate electrode contact with the skin, the thoracic circumference was measured in each patient before the EIT‐measurement. EIT measurements were performed continuously over a period of 5 min in a comfortable position for the respective patient (lying in the bed/horizontal position, lying in the bed/with upper body in upright position, or sitting in the bed/on a chair). EIT measurements were performed at two timepoints: (a) timepoint 1 (T1), directly before the CPT intervention; (b) timepoint 2 (T2), within 30 min after the CPT intervention. During CPT, which was performed by the respiratory physiotherapist, no EIT measurement was performed, and the electrode‐belt was detached. The patient was in the same body position (including rotation of the thorax) during T1 and T2 to provide comparable EIT data sets. In addition, electrode‐belt position was marked on the skin using adhesive tape or a skin marker to ensure that the electrode‐belt was kept in the same position when attached again at T2. The reference electrode (abdomen) was left attached and fixed with tape. EIT index calculation was performed using the PulmoVista500 software and a custom‐made software (MATLAB21a, The MathWorks Inc., Natick, MA, USA). The breathing pattern of every single patient was screened offline: 10 single breaths with a resting breathing pattern (without evidence of coughing, grunting, speaking, phonation) with a comparable ΔZ (tidal impedance variance) covering all breaths were selected at each timepoint (T1 and T2; see Figure S1).

In patients with respiratory support (oxygen supply via nasal cannula, CPAP via bi‐nasal prong or mask, or non‐invasive ventilation via full‐face mask), the settings of the support device were recorded (fraction of inspired oxygen [fiO_2_], liter of oxygen per minute, positive end‐expiratory pressure [PEEP in mbar]).

### 2.5 Chest Physiotherapy Interventions

Respiratory physiotherapists applied various techniques to increase pulmonary ventilation. Active breathing practices included: pursed lip breathing [1], forced expirations, thoracic expansion exercises, and breathing control [12]. Additionally, they used gentle exercises to support secretion clearance [13]. Other methods utilized as part of passive physiotherapy in critically ill or very young children were manual vibration, tapping/percussion, and positioning [2, 14]. Each session lasted approximately 30 min.

### 2.6 Subgroup Analysis, Outcome Parameters and Statistical Analysis

We did not perform a sample‐size and power calculation as this was a feasibility study and there are no comparable data sets. Demographic data and baseline characteristics are presented as median with interquartile range (IQR) or absolute number (n) with percentage. For comparison of mean values between timepoints of EIT measurements (T1 vs. T2) a general linear model with ANOVA for repeated measurements with Bonferroni‐Holm correction was used. For interpretation of categorical variables, the Fisher's exact test and chi‐square test were applied where applicable. A *p*‐value < 0.05 was considered significant. The statistical analysis was performed using statistical software (IBM SPSS Statistics for Windows, Version 27.0., IBM Corp, Armonk, NY).

## 3 Results

### 3.1 Patients’ Characteristics

**FIGURE 1:**
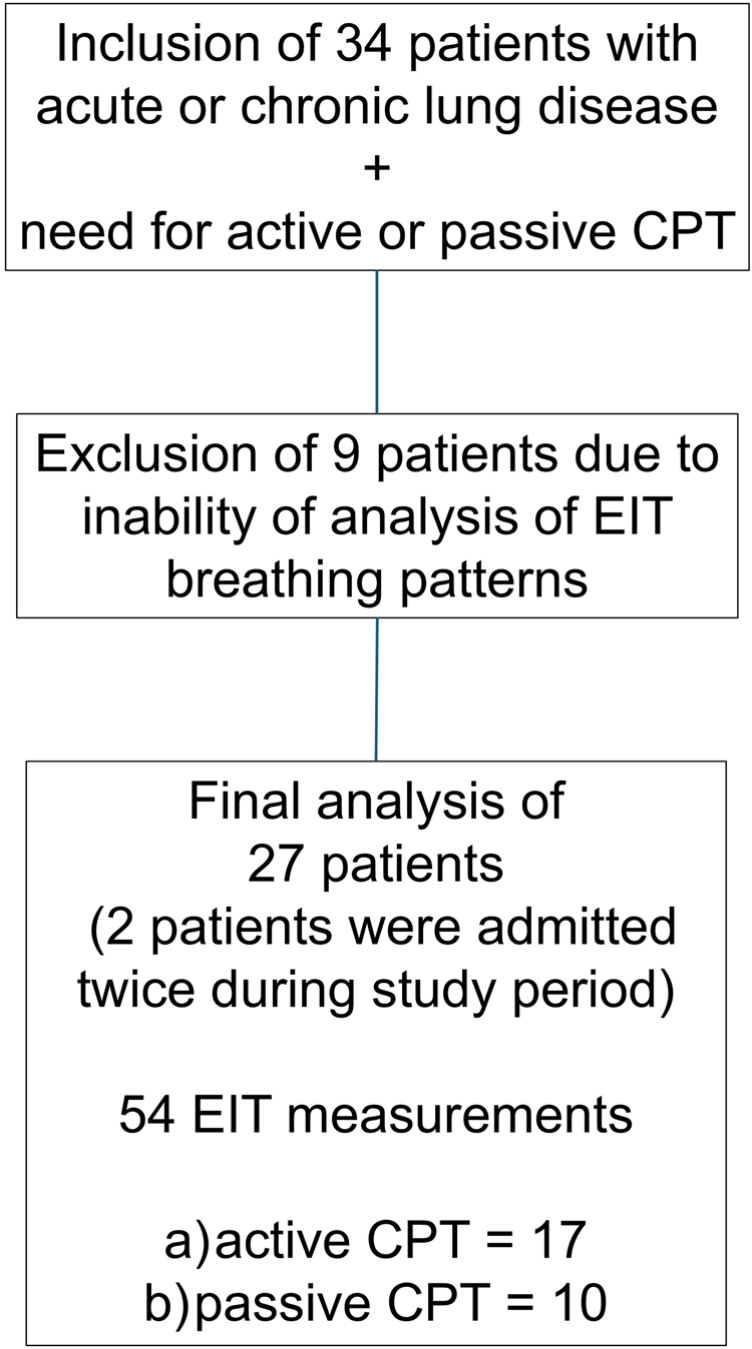
Flow‐chart of patients’ inclusion. [Color figure can be viewed at wileyonlinelibrary.com]

Overall, 34 patients were prospectively enrolled after signed written consent of the respective patient or legal guardian was obtained. Nine infants (aged 0–12 months, with body weight < 10 kg) were measured before and after CPT interventions, but EIT data could not be analyzed properly in the offline analysis due to very shallow breathing and tachypnea. Therefore, single breaths and impedance changes in the respective EIT measurements could not be detected appropriately using MATLAB and onboard software, and data sets were consecutively excluded. Data sets of 25 patients were finally analyzed. Two patients were enrolled at two different timepoints/hospital admissions and were measured twice, resulting in a total of 27 sets of EIT measurements (*n* = 54, see flow‐diagram in Figure 1). Detailed epidemiological data and patients’ characteristics are displayed in Table 1. Patients were subdivided into four different age groups (1–4) according to their age at time of EIT measurements (Table 1). The most common diagnoses in our cohort were: viral bronchitis/bronchiolitis, bronchopneumonia, lobar pneumonia, history of nARDS or pARDS with prolonged mechanical ventilation > 24 h, atelectasis/dystelectasis, pleural effusion (chylothorax or serous pleural effusion), and prophylactic CPT for serious underlying general conditions. Almost half of the patients were supported with non‐invasive respiratory support (Table 1). Two thirds of the patients received active CPT. In patients with radiological findings (chest X‐ray or lung ultrasound) before EIT measurements, these findings were equally localized in the left and right lung (50% each).

**TABLE 1:** Demographic and treatment data.

Variables	Patients *n* = 27 EIT‐measurements n= 54
**Demographic parameters**	
Age, years	3.0 (1/4.5)
Body weight, kg	12.2 (8.3/17)
Female sex, n (%)	10 (40)
Thoracic circumference, cm	48.5 (43.5/57)
**Respiratory support**	
a. Non‐invasive respiratory support, n (%) −. Oxygen nasal cannula −. HFNC −. CPAP b. No respiratory support, n (%)	14 (52) 8 (57) 5 (36) 1 (7) 13 (48)
**Body position**	
a. Supine b. Semi‐recumbent c. Sitting	9 (33) 12 (45) 6 (22)
**Acute respiratory diagnosis**	
a. Viral bronchitis, n (%) b. Bronchopneumonia, n (%) c. Lobar pneumonia, n (%) d. History of nARDS/pARDS, n (%) e. Pleural effusion, n (%) f. Prophylactic CPT, n (%)	3 (11) 14 (52) 3 (11) 1 (4) 1 (4) 5 (19)
**Localized pulmonary finding in chest X‐ray/lung ultrasound**	
a. Yes, n (%) b. No, n (%)	10 (37) 17 (63)
**Chest physiotherapy**	
a. active, n (%)	17 (63)
b. passive, n (%)	10 (37)

Abbreviations: CPAP, continuous positive airway pressure; CPT, chest physiotherapy; HFNC, high flow nasal cannula, nARDS, neonatal acute respiratory distress syndrome; pARDS, pediatric acute respiratory distress syndrome; Prophylactic CPT: prophylactic CPT for serious underlying general conditions such as immunodeficiency, heart defect, pre or post organ transplantation.

*Note:* Data are presented as absolute numbers with percentage or as median values with IQR (25/75).

### 3.2 EIT‐Findings

**FIGURE 2:**
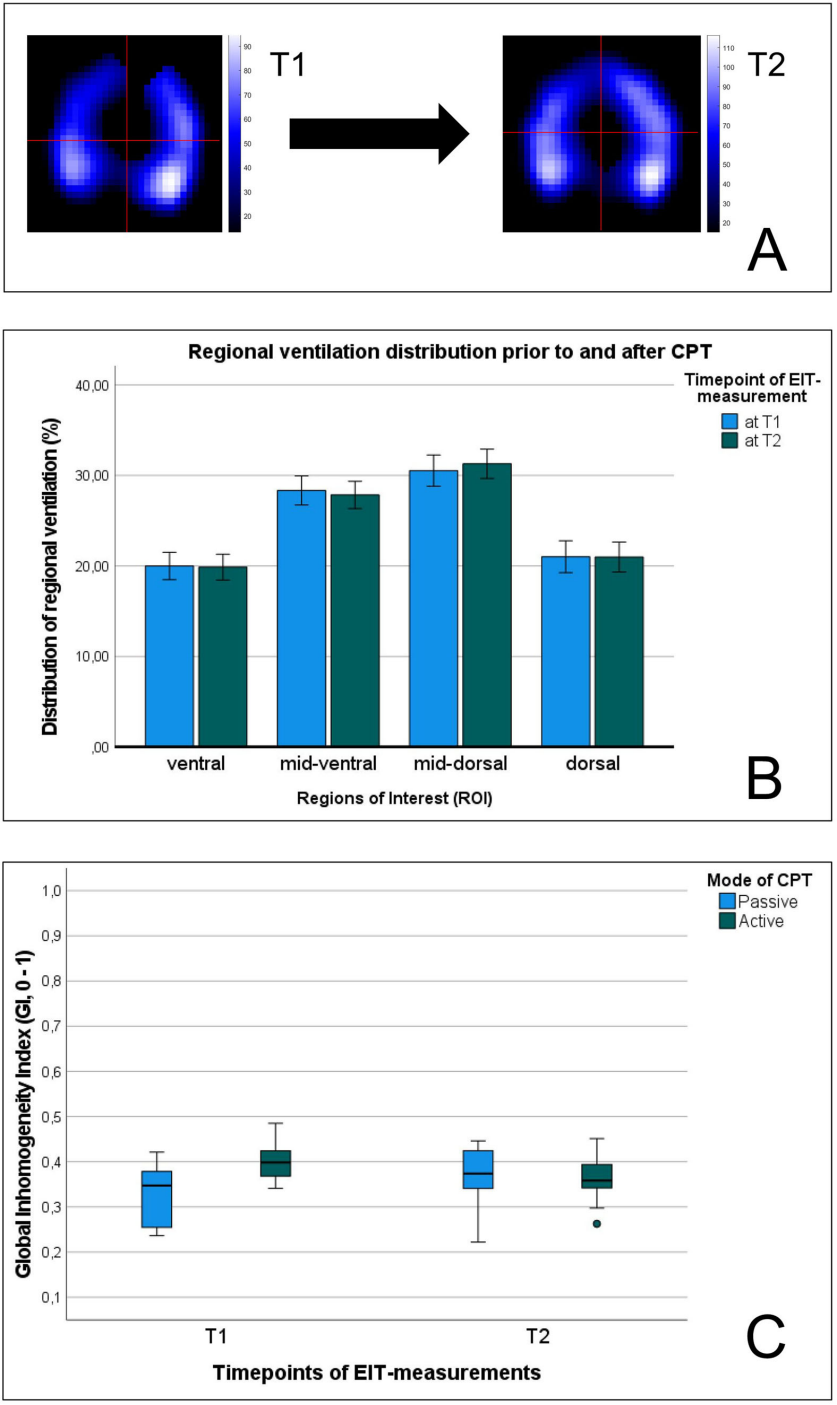
(A–C): (A) The distribution of the mean CoV (X + Y) is illustrated in panel A for respective patients before (T1) and after (T2) active CPT. (B) The regional ventilation distribution in four regions of interest (ROI, ventral‐dorsal) is illustrated for all patients at T1 and T2. (C) The Global Inhomogeneity Index (GI) is illustrated for the according subgroups (active vs. passive CPT) at T1 and T2. [Color figure can be viewed at wileyonlinelibrary.com]

The findings of EIT‐indices are displayed in Figures 1A–C and 2A/B, as well as in Table 2. In the overall cohort as well as between subgroups (CPT active/passive, and radiological finding yes/no) the mean CoV_X,_ and the CoV_Y_ were distributed equally at T1 and T2, without any significant differences between subgroups. The mean global inhomogeneity index (GI) decreased from 0.38 to 0.36 (∆GI: ‐6%, *p* = 0.939) in the overall cohort, with a significant decrease between T1 and T2 in patients with active (∆GI: ‐10%, *p* = 0.015), but not in patients with passive CPT (∆GI: +6%, *p* = 0.199).

**TABLE 2:** EIT‐indices at T1 and T2.

Variables	Patients‐Cohort (*N* = 27)
Horizontal Center of Ventilation (CoV_X_, 0–1, right‐left)	
at T1 (overall)	0.47 (0.09)
active CPT	0.47 (0.10)
passive CPT	0.46 (0.07)
at T2 (overall)	0.47 (0.08)
active CPT	0.47 (0.10)
passive CPT	0.46 (0.06)
Vertical Center of Ventilation (CoV_Y_, 0‐1, ventral‐dorsal)	
at T1 (overall)	0.51 (0.04)
active CPT	0.52 (0.04)
passive CPT	0.49 (0.05)
at T2 (overall)	0.51 (0.05)
active CPT	0.52 (0.06)
passive CPT	0.50 (0.05)

*Note:* Data are demonstrated as mean values with ± standard deviation. The timepoints of EIT‐measurements were classified as: T1 = before chest physiotherapy (CPT) and T2 = after CPT. The CoV describes the spatial distribution of lung ventilation in a horizontal (right‐left, CoV_X_) and vertical direction (dorsal‐ventral, CoV_Y_), where a value of 0.5 indicates a centered ventilation distribution in the horizontal and vertical direction.

**FIGURE 3:**
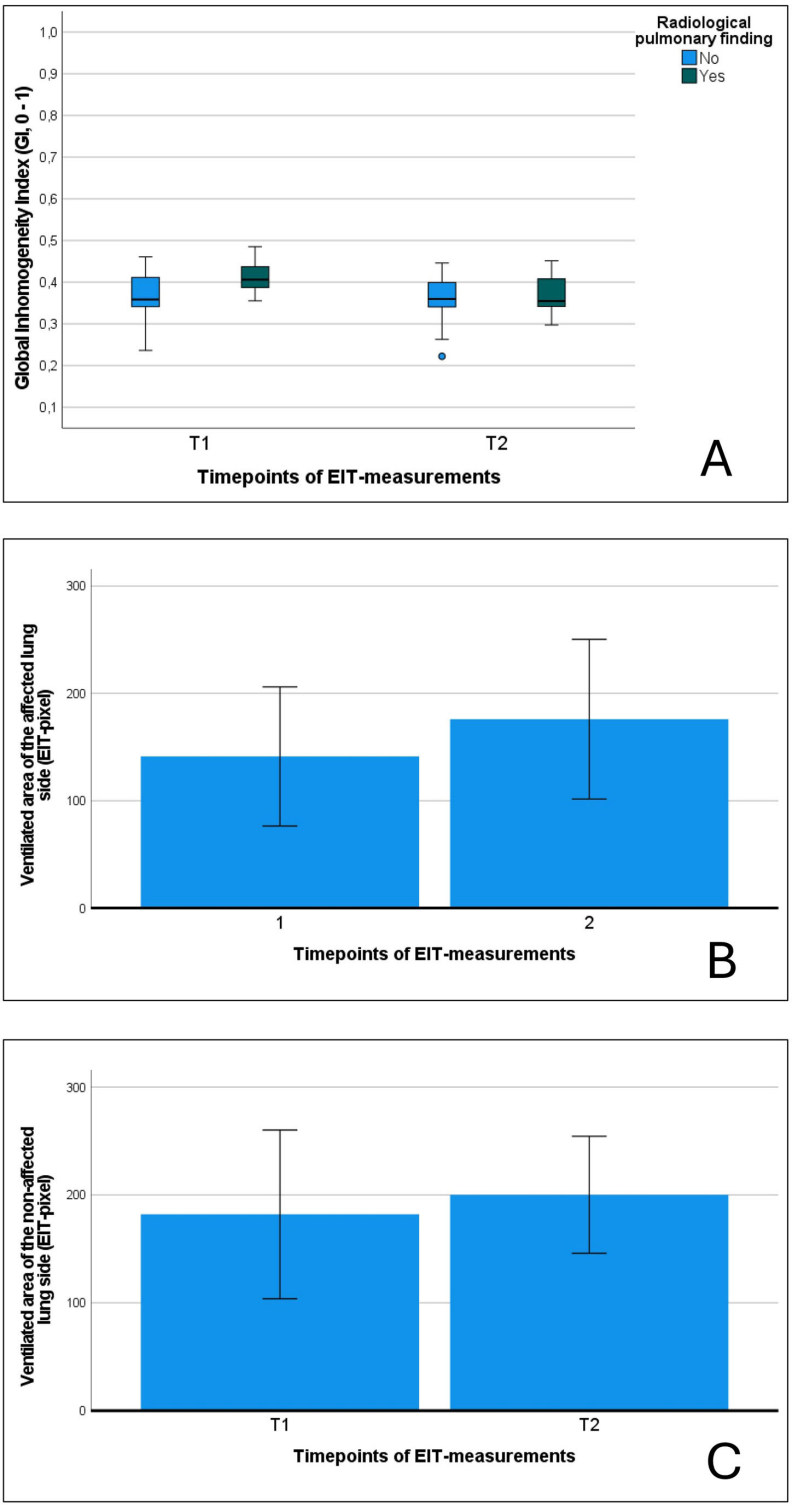
(A–C): (A) The Global Inhomogeneity Index (GI) is illustrated for the according subgroups (radiological pulmonary finding vs. no finding) at T1 and T2. (B) The regional ventilated lung area (EIT‐pixel) is illustrated for the affected lung side (radiological pulmonary finding) (B) and the non‐affected lung side (C). [Color figure can be viewed at wileyonlinelibrary.com]

We further subclassified patients regarding radiological findings in chest X‐ray or lung/pleural ultrasound. In patients with a positive radiological finding (pneumonia, atelectasis/dystelectasis; *n* = 10) the GI decreased after the CPT intervention (0.42 vs. 0.37, *p* = 0.093; Figure 3A). Additionally, the ventilated lung area (EIT‐pixel; right or left lung; calculated for the affected lung side) increased after the CPT intervention (all active CPT; 141 vs. 176; *p* = 0.128; Figure 3B). The ventilated lung area of the non‐affected lung side in these patients also increased (182 vs. 200, *p* = 0.735; Figure 3C). When comparing patients with and without respiratory support (HFNC, CPAP, or NIV) no significant differences were found in GI, or CoV_X/Y_ at T1 or T2. Body position during CPT did not significantly influence measurements of EIT indices.

### 3.3 Respiratory Physiotherapist Questionnaire

We asked all respiratory physiotherapists to participate in a survey (Table 3) about the EIT‐feasibility trial, with focus on practicability of EIT in pediatric patients with need for CPT and expectations. Overall, eight respiratory physiotherapists participated and answered to 11 items. None of the respiratory physiotherapists had any experience with EIT before the study. The majority reported positive feedback and further interest in using and including EIT for future research or practice. Most of the physiotherapists felt that EIT‐procedure was time consuming but did not require much patient cooperation. When the EIT‐procedure was considered as an influencing factor on the manual (active/passive) intervention of the respiratory physiotherapists (in 25%) the influence by the EIT‐procedure was felt to be positive (100%). Nevertheless, one person felt that the EIT‐procedure negatively influenced patients’ compliance in the respiratory intervention. Overall, 50% felt that the EIT‐procedure was suitable apart from the smallest age groups (< 2 years and 2–4 years).

**TABLE 3:** Respiratory physiotherapist survey.

Questions/Items	Answers (*N* = 8 respiratory physiotherapists)
Have you had any previous contact with the use of electrical impedance tomography (EIT) as a new technique for bedside lung monitoring?	no: 8/8 (100); yes: 0/8 (0)
Did the EIT measurement before and after the physiotherapy application influence the therapy measures you carried out?	no: 5/8 (62); yes: 3/8 (38)
if YES: Was the influence rather positive or rather negative?	positive: 3/3 (100)
Did the EIT measurement influenced patient compliance?	no: 6/8 (75); yes: 2/8 (25)
if YES: Was the influence rather positive or rather negative?	positive: 1/2 (50), negative: 1/2 (50)
Did you find the individual EIT measurements rather short or rather time‐consuming? (from very time‐consuming [□ 1–□ 5] to very short)	1: 1/8 (13); 2: 3/8 (38); 3: 3/8 (38); 4: 1/8 (13); 5: 0/8 (0)
Did you find the EIT process to be more resource‐intensive (e.g., labor, consumables, etc.) or more resource‐saving in terms of benefits? (from very resource‐consuming [□ 1–□ 5] to very resource‐saving)	1: 0/8 (0); 2: 5/8 (62); 3: 3/8 (38); 4: 0/8 (0); 5: 0/8 (0)
In your opinion, did EIT require much or little patient cooperation? (from very much [□ 1–□ 5] to very little)	1: 0/8 (0); 2: 0/8 (0); 3: 3/8 (38); 4: 0/8 (0); 5: 5/8 (62)
Have you received feedback from patients and/or their guardians regarding the EIT measurement?	no: 5/8 (62); yes: 3/8 (38)
If YES: was the feedback you received from patients and/or their guardians about the procedure positive or negative?	positive: 2/3 (67), negative: 1/3 (33)
Could you generally imagine using the EIT as a biofeedback tool in your physiotherapy interventions in the future after adequate instruction and with the support of other staff? (from not at all [□1–□ 5] to very good)	1: 0/8 (0); 2: 2/8 (25); 3: 0/8 (0); 4: 4/8 (50); 5: 2/8 (25)
Apart from what age group could you imagine using EIT as a biofeedback tool? (apart from 1) < 2 years, 2) 2–4 years, 3) 5–6 years, 4) 6–12 years, 5) > 12 years)	1: 3/8 (38); 2: 1/8 (13); 3: 2/8 (25); 4: 2/8 (25); 5: 2/8 (25)
Has EIT aroused your interest and could you therefore in principle imagine taking part in further training in the use of EIT as a biofeedback tool? (from not at all interested [□ 1–□ 5] to very interested)	1: 1/8 (13); 2: 0/8 (0); 3: 0/8 (0); 4: 5/8 (63); 5: 1/8 (13)
When you weigh up the costs and benefits, do you think the ubiquitous use of EIT is realistic for physiotherapists in everyday clinical practice? (from unrealistic [□ 1–□ 5] to very realistic)	1: 0/8 (0); 2: 0/8 (0); 3: 4/8 (50); 4: 4/8 (50); 5: 0/8 (0)

*Note:* Data of the answers are presented as absolute numbers (n) with %.

## 4 Discussion

This prospective feasibility study is the first study to evaluate the use of EIT to monitor the regional ventilation distribution after CPT in spontaneously breathing pediatric patients. The key findings are as follows: in patients with an active CPT intervention the GI significantly decreased after the CPT intervention, whereas this could not be observed in patients after passive CPT. In patients with localized radiological pulmonary findings the ventilation lung area (EIT‐pixel) of the affected lung side increased after the CPT intervention. EIT measurements in spontaneously breathing patients < 10 kg might be challenging due to shallow spontaneous breathing patterns potentially impairing EIT interpretation.

EIT has been studied for more than 20 years as an innovative monitoring technique that enables radiation free assessment of regional ventilation distribution in patients with pulmonary disease. However, data in pediatric patients are still scarce and this technique is still trying to find its way into clinical practice. As the assessment of spontaneous breathing is challenging in pediatric patients, apart from techniques such as spirometry, the EIT offers manifold advantages as bedside monitoring technique. Particularly in view of the ongoing debate whether CPT is effective in pediatric patients (according to Cochrane reviews from recent years) the EIT technique could help researchers to provide more insights into pulmonary function in clinical trials [1, 3, 4, 5]. While the 2016 Cochrane review reported no significant benefit of CPT for severe bronchiolitis in children aged 0–24 months and outlined potential risks of transient airway complications, it emphasized the need for further studies, particularly exploring slow passive expiratory techniques in mildly to moderately affected RSV‐positive patients [4]. Our feasibility study potentially adds new information to this field, showing improvements in regional lung ventilation and distribution in children with moderate impairment and predominantly viral infections.

In a secondary analysis of a RCT in children with invasive mechanical ventilation, McAlinden et al. reported improvements in the regional ventilation distribution after CPT, as evaluated with EIT‐indices [10]. The authors reported a significant increase of the end‐expiratory lung volume (EELV), as a measure of the functional residual capacity, and an improvement of the CoV as well as GI after CPT as compared to the routine airway clearance (endotracheal suctioning). These findings are in line with our data, as the authors also reported an improvement of regional ventilation distribution after CPT. However, the study results from McAlinden et al. and our results cannot be compared without caution, as the patient cohorts differed markedly.

The increase of the GI Index seen in some patients of the cohort suggests an increasing lung inhomogeneity, potentially resulting from poorly aerated or collapsed lung regions [15]. This finding might go in line with the observation of clinical trials, that CPT may induce recruitment of atelectatic lung regions with the potential risk of redistributing secretions into smaller airways with the potential re‐induction of atelectasis and bronchospasms.

Our findings point out that EIT is potentially helpful as an enhanced targeted therapy in localized ventilation disorders. EIT revealed noteworthy improvements in the regional ventilation distribution in previously identified lung areas with radiological pulmonary findings (e.g., infiltrates, atelectasis/dystelectasis), underscoring its potential role in optimizing CPT interventions. Li et al.‘s study corroborates this, showing that EIT helps personalize CPT by improving ventilation distribution [9]. In their feasibility trial Li and colleagues for the first time presented a flow‐algorithm how to incorporate EIT in daily clinical practice in patients with pulmonary disorders and the need for CPT. Similarly, Davies et al. (2019) reported successful lung re‐expansion and accelerated weaning from ventilation in pediatric patients with RSV pneumonia and lung collapse, following EIT‐guided adjustments in positioning and therapy [11]. These studies, along with our findings, suggest EIT's crucial role in tailored respiratory care and individualized treatment.

In their study McAlinden et al. observed an improvement in regional ventilation distribution of dependent lung areas following manual CPT, as evidenced by a shift in the CoV_Y_ towards dependent dorsal lung regions [10]. According to our data we similarly revealed a trend towards improvement with a more centered ventilation distribution in children after CPT. In children with active CPT the CoV_Y_ shifted towards ventral, nondependent lung regions (0.52–0.5), whereas in children with passive GPT the CoV_Y_ shifted towards dorsal, dependent lung regions (0.48–0.5). As CoV_Y_ values only differed slightly between subgroups it is challenging to distinguish where these differences may derive from. Differences between patients can certainly be explained by the underlying condition, type of infection, or the side and region of affected lung area.

The more moderate changes across all indices observed in children with passive CPT, compared to those receiving active CPT, align with the findings of Gomes et al [2]. Gomes et al. noted that children with either severe or mild conditions often do not benefit as much from respiratory therapy techniques, whereas those with moderate conditions show greater improvements [2]. In our study, the passively treated children were generally in more severe clinical conditions, potentially explaining their only moderate responses and reflecting the reduced efficacy of passive CPT. In contrast, children with active CPT, who were in better overall health conditions, exhibited more pronounced changes due to their greater responsiveness to CPT. Also, age appears to play an important role, as in infants and toddlers active CPT is not possible due to limitations of verbal communication and motor skills. Additionally, the type of respiratory support could have a potential influence on effects after the CPT. For our data set we could exclude that EIT indices significantly differed according to the type of respiratory support.

### 4.1 Future Directions

The use of EIT for monitoring respiratory function offers promising opportunities, particularly in terms of biofeedback and personalized respiratory therapy for children. Our study lays the foundation for future research exploring EIT as a precise tool to monitor and enhance CPT. EIT could be utilized to provide real‐time feedback to children receiving active CPT about their breathing patterns, encouraging active participation, and increasing their awareness of their respiratory function (see Figure S2). This would be a valuable step towards personalized therapy, considering each child's unique respiratory physiology to optimize treatment outcomes. The consensus expert statements from the TREND group and as stated by Scaramuzzo et al. also highlight EIT's current potential to precisely monitor and optimize individual ventilation needs, further supporting its future role in personalized respiratory therapy [8, 16]. Furthermore, EIT might provide important bedside information for the attending physician and respiratory physiotherapists in patients with “visible” mismatch of regional ventilation (e.g., due to atelectasis or pneumonia).

### 4.2 Study Limitations

Our study had several limitations. First, this is a single‐center study with a comparatively small sample size of ultimately 25 participants. The EIT technique has some limitations of its own: (a) EIT provides regional longitudinal information of a certain area of the lung, but not for the global lung, (b) long‐term effects of CPT could not be evaluated, and effects might be under‐ or overestimated, as we measured only once after the intervention, (c) a misalignment in the rotation and position of the EIT belt, and body position at T1 and T2 might cause bias in the data interpretation. Additionally, the selection of 10 single breaths might lead to misinterpretation of EIT indices due to observer dependent breath selection. The wide age range of 0–17 years may have contributed to varied outcome measures that are difficult to generalize. Additionally, the effectiveness of CPT may have varied depending on the individual therapist administering the treatment. Therefore, a longitudinal prospective study is planned to compare our preliminary findings in bigger subgroups and to provide data from patients with invasive mechanical ventilation.

## 5 Conclusion

In conclusion, our study demonstrates that it is feasible to monitor the effect of CPT using EIT in children with pulmonary diseases. EIT‐guided CPT might play a future role in improving regional ventilation during spontaneous breathing in these patients. Looking ahead, EIT could become a central bedside tool in a personalized respiratory physiotherapy, not only for monitoring purposes but also as a feedback mechanism to maximize therapeutic efficacy and achieve better long‐term outcomes.

## Author Contributions

**Johanna Moersdorf:** conceptualization, investigation, writing–original draft, methodology, validation, visualization, formal analysis, data curation, software. **Thomas Muders:** methodology, writing–review and editing, validation, investigation, software. **Christian Putensen:** methodology, writing–review and editing, validation. **Ulrike Heller:** investigation, conceptualization, writing–review and editing, methodology. **Andreas Mueller:** supervision, methodology, validation, writing–review and editing, conceptualization. **Lukas Schroeder:** project administration, formal analysis, supervision, writing–original draft, investigation, conceptualization, methodology, validation, software.

## Consent

Patients were prospectively enrolled in the study after informed written consent was obtained from the parents or legal representative. In our manuscript we use an image (Figure S2) with possible identification of study participants (patients) or health care workers (attending physician or nursing stuff). Therefore, we obtained a written consent for publication purposes before manuscript submission from the illustrated persons and legal guardians. During the preparation of this work the author(s) did not used any generative AI or AI‐assisted technology.

## Conflicts of Interest

The authors declare no conflicts of interest.

## Supporting information


**Online Supporting material: Figure 1**: Breathing patterns measured with the EIT‐belt over a period of 2 minutes in a pediatric patient prior to CPT. 10 single breaths in a resting breathing pattern were selected for further offline analysis for EIT‐indices.

**Online Supporting material: Figure 2**: The image illustrates a potential future scenario for the interactive use of EIT‐monitoring in a pediatric patient (middle) during active chest physiotherapy with the respiratory physiotherapist (right) and the attending physician (right). With the possibility of the visualization of ventilation distribution as well as in‐ and expiration the pediatric patient can be motivated to perform breathing exercises.

## Data Availability

The data that support the findings of this study are available from the corresponding author upon reasonable request.
