# Supplementary figures and images for: Monitoring of Regional Ventilation Distribution Using Electrical Impedance Tomography in Pediatric Patients With Chest Physiotherapy—A Feasibility Study
Source: Pediatr Pulmonol. 2025 Feb 26;60(2):e71014. doi: 10.1002/ppul.71014 (PMC11863537; doi:10.1002/ppul.71014)

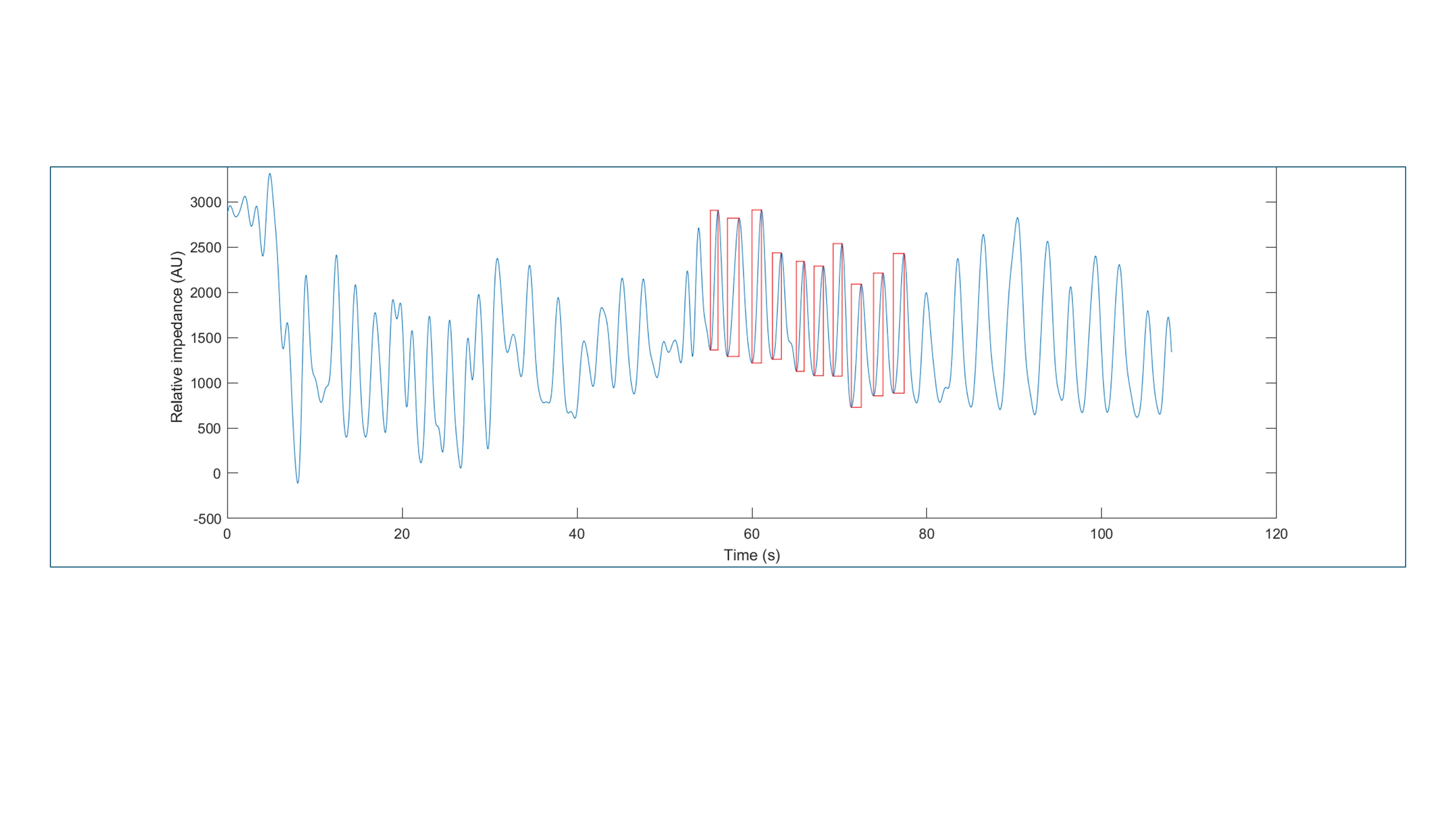

Supplement: Supplementary file 1 — Online Supporting material: Figure 1: Breathing patterns measured with the EIT‐belt over a period of 2 minutes in a pediatric patient prior to CPT. 10 single breaths in a resting breathing pattern were selected for further offline analysis for EIT‐indices. [file PPUL-60-0-s002.jpg]

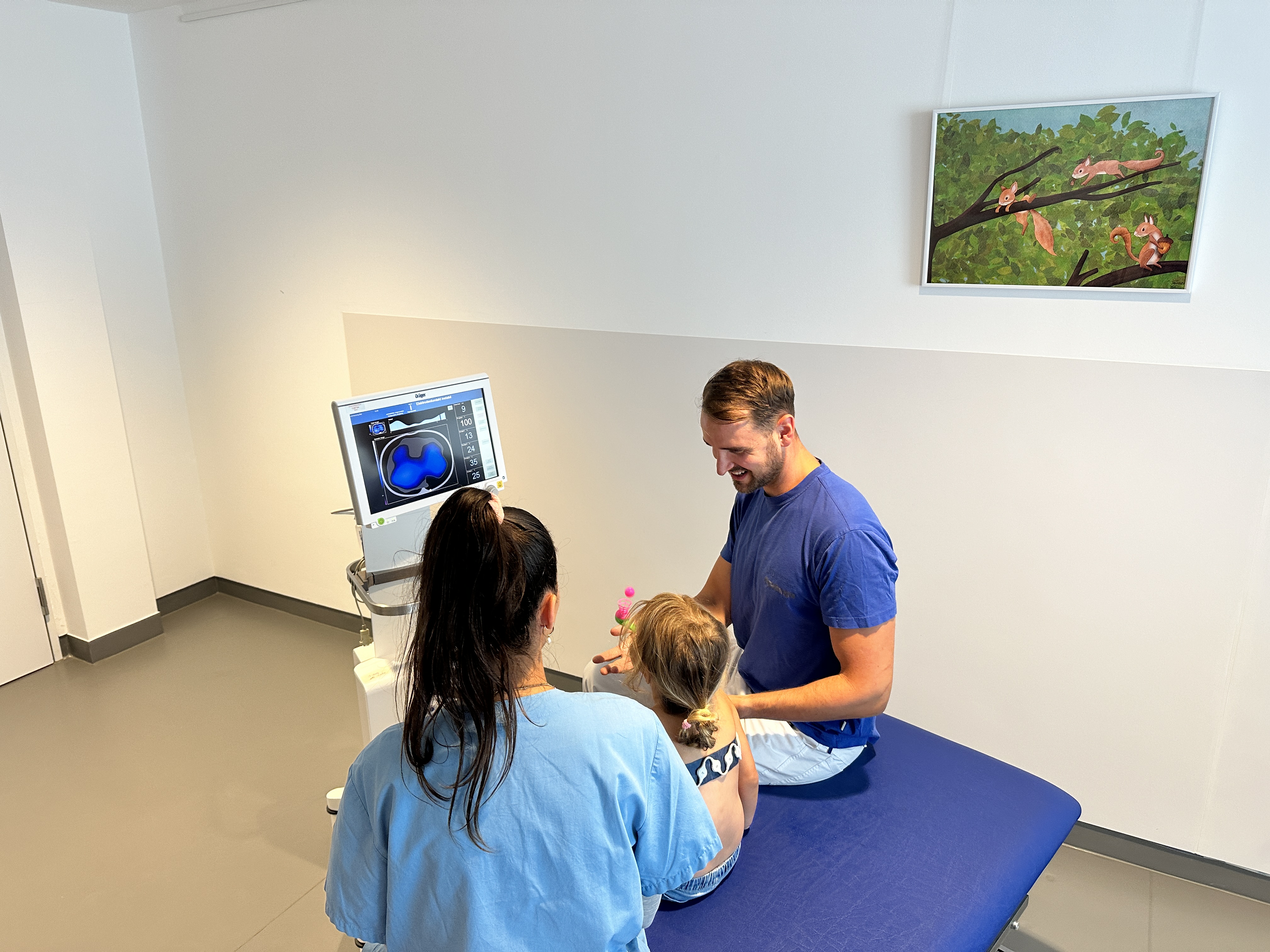

Supplement: Supplementary file 2 — Online Supporting material: Figure 2: The image illustrates a potential future scenario for the interactive use of EIT‐monitoring in a pediatric patient (middle) during active chest physiotherapy with the respiratory physiotherapist (right) and the attending physician (right). With the possibility of the visualization of ventilation distribution as well as in‐ and expiration the pediatric patient can be motivated to perform breathing exercises. [file PPUL-60-0-s001.JPG]
